# The activity of pregnancy-associated plasma protein A (PAPP-A) as expressed by immunohistochemistry in atherothrombotic plaques obtained by aspiration thrombectomy in patients presenting with a ST-elevation myocardial infarction: a brief communication

**DOI:** 10.1186/1477-9560-8-1

**Published:** 2010-01-27

**Authors:** Trygve Brügger-Andersen, Leif Bostad, Dagny Ann Sandnes, Alf Inge Larsen, Vernon VS Bonarjee, Ståle Barvik, Tor Melberg, Dennis WT Nilsen

**Affiliations:** 1 Institute of Medicine, University of Bergen, 5021 Bergen, Norway; 2 Department of Medicine, Stavanger University Hospital, 4068 Stavanger, Norway; 3 Institute of Surgery, University of Bergen, 5021 Bergen, Norway; 4 Department of Pathology, Haukeland University Hospital, Bergen, Norway; 5 Section for Pathology, The Gade Institute, University of Bergen, 5021 Bergen, Norway

## Abstract

**Background:**

The expression of pregnancy-associated plasma protein A (PAPP-A) was identified by immunohistochemistry (IHC) in culprit atherothrombotic plaque specimens harvested from patients admitted with ST-segment elevation myocardial infarction (STEMI).

**Methods:**

The atherothrombotic samples were collected from a consecutive cohort consisting of 20 individuals admitted with STEMI to Stavanger University Hospital, Norway, from 2005-2006, presenting angiographically with an acute thrombotic occlusion of a coronary artery characterized by TIMI flow 0. The atherothrombotic plaques were obtained by aspiration thrombectomy during percutaneous coronary intervention within 12 hours from the onset of symptoms and prepared for IHC analysis.

**Results:**

In the IHC analysis staining for PAPP-A occurred in the extracellular matrix of the plaques and no evidence of staining for PAPP-A was found in the thrombi.

**Conclusion:**

Our results indicate that in vivo PAPP-A is strongly expressed in atherothrombotic plaques harvested from patients admitted with STEMI, as documented by IHC.

**Trial registration:**

biobankregisteret@fhi.no1846

## Background

Pregnancy-associated plasma protein A (PAPP-A) is a zinc-binding matrix metalloproteinase that can be detected in the blood of patients with acute coronary syndromes (ACS) [1,2]. There is histological evidence, using specific monoclonal antibodies, that PAPP-A is abundantly expressed in both eroded and ruptured coronary plaques, but not in stable plaques, in patients who have died suddenly of cardiac causes. Furthermore, accumulating evidence suggests that PAPP-A may play a pivotal role in the development of atherosclerosis and subsequent plaque instability in ACS patients [1].

In a prior study we have assessed the immediate effects of coronary reperfusion procedures on the plasma concentrations of PAPP-A in patients admitted with ACS and ST-elevation myocardial infarction (STEMI) [3]. However, existing data does not allow us to define the exact role of PAPP-A in plaque disruption. Although, this metalloproteinase has been shown in earlier studies to be expressed in ruptured plaques, these results were limited by the fact that the histological samples were collected postmortem [1]. Therefore, we wanted to identify the expression of PAPP-A by immunohistochemistry (IHC) in culprit atherothrombotic plaque specimens harvested from patients admitted with STEMI.

## Methods

The atherothrombotic plaques, consisting of a mixture of unstable plaques and intra-coronary occlusive and mural thrombi, were obtained by aspiration thrombectomy during percutaneous coronary intervention (PCI) from 20 subjects admitted with STEMI. These patients received 5.000-7.500 IU of unfractionated heparin during the procedure. The subjects belonged to a consecutive cohort admitted within 125 (30-720) minutes [median (range)] from the onset of symptoms to Stavanger University Hospital, Norway, from 2005-2006. They presented angiographically with an acute thrombotic occlusion of a coronary artery characterized by TIMI flow 0. The atherothrombotic specimens with plaque fragments were collected during the PCI procedure and immediately placed into a tissue fixative consisting of formalin.

**Figure 1:**
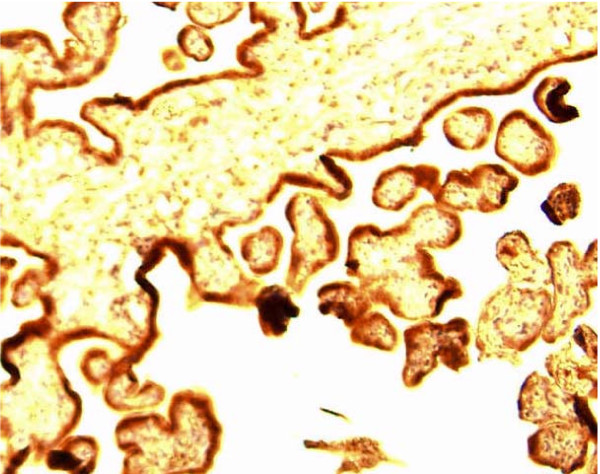
**Immunohistochemistry of placental tissue (positive control)**. The trophoblastic layer is strongly expressing PAPP-A (reddish brown staining).

The formalin treated samples were first stored one hour in 70% ethanol, and then embedded in paraffin. Briefly, 4-μm-thick sections from the paraffin blocks were air-dried on superfrost slides, and then frozen until use. Sections from all the blocks were stained with haematoxylin and eosin for general orientation and identification of tissues and structures. The slides for IHC were deparaffinised with toluene, rehydrated in ethanol, boiled in 10 mM Citratebuffer pH 6.0 and TBS-0.025% Tweenbuffer pH 7.6, and incubated with the primary antibody; the polyclonal rabbit anti-human Pregnancy Associated Plasma Protein A: Ig fraction (A0230 Dako), Denmark. For IHC detection and visualisation we used the DakoCytomation EnVision+ System HRP, Rabbit K4010, Dako, Denmark. Furthermore, histological specimens from surgically removed human placenta tissue were used as positive controls (Figure 1).

## Results

**Figure 2:**
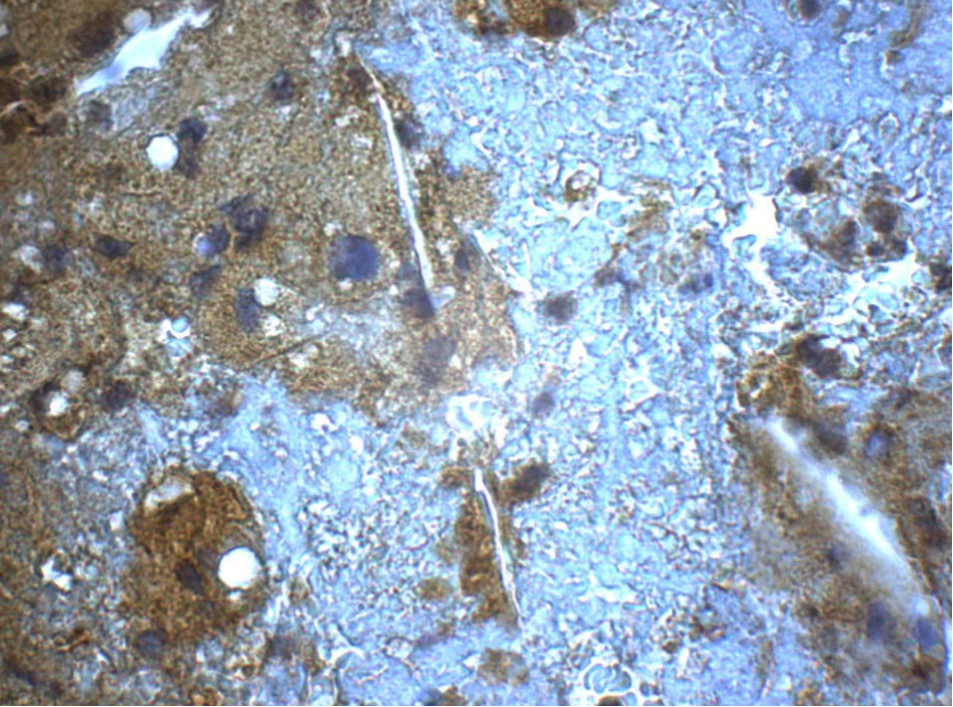
**Immunohistochemistry of sampled atherothrombotic material**. Atheromatous area showing diffuse immunoreactivity to PAPP-A (reddish brown staining). The staining is negative in the thrombus (white-blue area).

**Figure 3:**
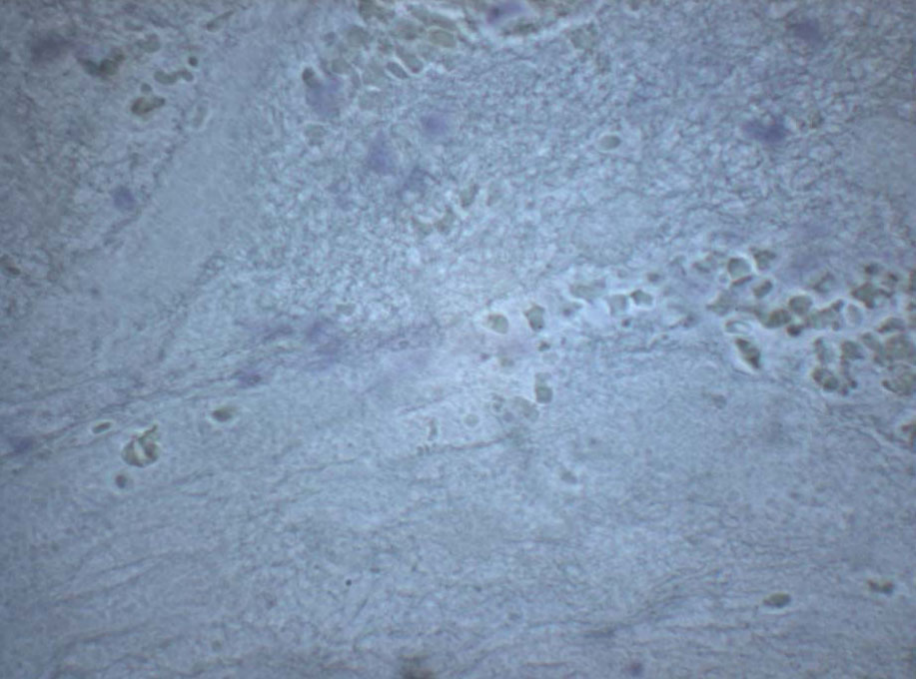
**Sampled thrombus without atheromatous material from the plaque**. Immunohistochemistry of proteinaceous material containing fibrin, platelets, erythrocytes and white blood cells without PAPP-A expression.

Seven samples were not suitable for evaluation because of technical reasons mainly due to insufficient material collected at the site of coronary occlusion. In three of the 13 remaining samples, plaque components were characterized by necrotic material with remnants of cholesterol-crystals (Figure 2). Inhomogeneously structured conglomerates of proteinaceous material containing fibrin, platelets, erythrocytes, and scattered white blood cells, were consistent with a thrombus (Figure 3). PAPP-A expression was demonstrated in the extracellular matrix of the plaques as illustrated by figure 2. There was no staining for PAPP-A in the thrombi (Figure 3).

## Discussion

Our results suggest a relation between ACS and the expression of PAPP-A in atherothrombotic plaques. This finding is in accordance with the study conducted by Bayes-Genis and colleagues [1], whereas, immunohistochemical analysis by Rossen et al. [4] failed to identify PAPP-A in atherosclerotic plaques from ACS patients. The discrepancies reported may be explained by the difference in the molecular nature of PAPP-A originating from placenta as compared to arterial atheromatous plaques. Thus, some assays developed for the placental form may also react against the plaque-associated form, whereas others will not, depending on the nature of the antibodies in the test systems [5]. Furthermore, it has been shown that heparin effectively competes for the binding site of PAPP-A on cell surfaces [6]. In addition, results from an interesting animal study documented that heparin facilitates the detachment of PAPP-A from the vessel wall [7]. Therefore, heparin administration during STEMI could explain the lack of PAPP-A staining in the thrombi. Despite these controversies, it cannot be excluded that the plaques may play a role as a reservoir for PAPP-A release following PCI [3]. However, the exact mechanistic role of this marker needs to be clarified.

## Conclusion

Our results indicate that in vivo PAPP-A is strongly expressed in atherothrombotic plaques harvested from patients admitted with STEMI, as documented by IHC.

## Competing interests

The authors declare that they have no competing interests.

## Authors' contributions

TBA had substantial contributions to conception and design and interpretation of IHC analysis and writing the manuscript. LB had substantial contributions to design and interpretation of IHC analysis. DAS carried out IHC analysis. AIL, VVSB, SB and TM had contribution to sampling. DWTN had substantial contributions to conception, sampling, design and writing the manuscript. All the authors have read and approved the final manuscript.

## Sources of support

Western Norway Regional Health Authority, Department of Pathology, Haukeland University Hospital, Norway

## References

[B1] Bayes-Genis A Conover CA Overgaard MT Bailey KR Christiansen M Holmes DR Jr Virmani R Oxvig C Schwartz RS Pregnancy-associated plasma protein A as a marker of acute coronary syndromes N Engl J Med 2001 345 1022 9 10.1056/NEJMoa003147 11586954

[B2] Boldt HB Overgaard MT Laursen LS Weyer K Sottrup-Jensen L Oxvig C Mutational analysis of the proteolytic domain of pregnancy-associated plasma protein-A (PAPP-A): classification as a metzincin Biochem J 2001 358 359 67 10.1042/0264-6021:3580359 11513734 PMC1222068

[B3] Brugger-Andersen T Hetland O Ponitz V Grundt H Nilsen DW The effect of primary percutaneous coronary intervention as compared to tenecteplase on myeloperoxidase, pregnancy-associated plasma protein A, soluble fibrin and D-dimer in acute myocardial infarction Thromb Res 2007 119 415 21 10.1016/j.thromres.2006.03.009 16650886

[B4] Rossen M Iversen K Teisner A Teisner B Kliem A Grudzinskas G Optimisation of sandwich ELISA based on monoclonal antibodies for the specific measurement of pregnancy-associated plasma protein (PAPP-A) in acute coronary syndrome Clin Biochem 2007 40 478 84 10.1016/j.clinbiochem.2006.11.025 17316591

[B5] Qin QP Kokkala S Lund J Tamm N Qin X Lepantalo M Pettersson K Immunoassays developed for pregnancy-associated plasma protein-A (PAPP-A) in pregnancy may not recognize PAPP-A in acute coronary syndromes Clin Chem 2006 52 398 404 10.1373/clinchem.2005.058396 16423908

[B6] Laursen LS Overgaard MT Weyer K Boldt HB Ebbesen P Christiansen M Sottrup-Jensen L Giudice LC Oxvig C Cell surface targeting of pregnancy-associated plasma protein A proteolytic activity. Reversible adhesion is mediated by two neighboring short consensus repeats J Biol Chem 2002 277 47225 34 10.1074/jbc.M209155200 12370176

[B7] Terkelsen CJ Oxvig C Norgaard BL Glerup S Poulsen TS Lassen JF Moller HJ Thuesen L Falk E Nielsen TT Andersen HR Temporal course of pregnancy-associated plasma protein-A in angioplasty-treated ST-elevation myocardial infarction patients and potential significance of concomitant heparin administration Am J Cardiol 2009 103 29 35 10.1016/j.amjcard.2008.08.027 19101225

